# Occupational Solvent Exposure and Brain Function: An fMRI Study

**DOI:** 10.1289/ehp.1002529

**Published:** 2011-02-04

**Authors:** Cheuk Ying Tang, David M. Carpenter, Emily L. Eaves, Johnny Ng, Nimalya Ganeshalingam, Clifford Weisel, Hua Qian, Gudrun Lange, Nancy L. Fiedler

**Affiliations:** 1 Department of Radiology, and; 2 Department of Psychiatry, Mount Sinai School of Medicine, New York, New York, USA; 3 Department of Environmental and Occupational Medicine; 4 Department of Psychiatry, and; 5 Department of Radiology, UMDNJ-New Jersey Medical School, Newark, New Jersey, USA

**Keywords:** brain function, fMRI, solvent exposure

## Abstract

Background: Deficits in cognitive function have been demonstrated among workers chronically exposed to solvents, but the neural basis for these deficits has not been shown.

Objectives: We used functional magnetic resonance imaging (fMRI) to compare pathophysiological changes in brain function between solvent-exposed and control workers.

Methods: Painters, drywall tapers, and carpenters were recruited from the International Union of Painters and Allied Trades, District Council 9 in New York City and District Council 21 in Philadelphia, Pennsylvania, and from the Carpenters Union in New Jersey. Twenty-seven solvent-exposed and 27 control subjects of similar age, education, and occupational status completed the N-Back working memory test during fMRI. After controlling for confounders (age; lifetime marijuana, cocaine, and alcohol use; blood lead; symptoms of depression; verbal intelligence), voxelwise group analysis and regional activation levels were compared and then correlated with an index of lifetime solvent exposure.

Results: Solvent-exposed workers’ performance on the N-Back was significantly worse than that of controls. Activation of the anterior cingulate, prefrontal, and parietal cortices—areas serving working memory function and attention—was also significantly lower for solvent-exposed workers relative to controls. After controlling for confounders, we observed a negative correlation between lifetime solvent exposure and activation in these same regions among the solvent-exposed workers.

Conclusions: This study is one of the few to document neural structures affected by exposure to solvents. Our findings provide a biological mechanism for the neurobehavioral deficits in working memory and attention that have previously been reported by other groups studying the effects of chronic exposure to solvents. These imaging markers, which are consistent with the neurobehavioral measures in our subject population, are consistent with altered brain pathology caused by prolonged exposure to solvent mixtures during construction work.

Workers chronically exposed to solvent mixtures frequently exhibit significantly compromised attention, processing speed, and working memory relative to demographically similar unexposed controls (Baker 1994; Benignus et al. 2005; Mikkelsen 1997). Although anatomical studies using magnetic resonance imaging (MRI) and computed tomography (CT) have detected structural changes, these have typically been seen only in subjects with severe solvent exposures, such as workers diagnosed with chronic toxic encephalopathy (Ellingsen et al. 1993; Thuomas et al. 1996) or organic solvent abusers (Filley et al. 1990; Rosenberg et al. 1988; Unger et al. 1994; Yamanouchi et al. 1995). Moreover, many of these studies suffered from poor characterization of exposure, confounding, inability to determine dose response, and biased subject selection (Ridgway et al. 2003). Other studies involving workers with varying levels and durations of exposure have also reported no cognitive correlations (Gericke et al. 2001; Seeber et al. 2009).

In the absence of a clear neurobiological basis for compromised cognitive performance, several investigators have questioned the validity of solvent-induced cognitive impairment (Gericke et al. 2001). However, cognitive changes observed in solvent-exposed subjects may be due to neurochemical alterations that can be detected with functional imaging. For example, in a study by Haut et al. (2000), positron emission tomography (PET) scans revealed that subjects diagnosed with chronic solvent encephalopathy (*n* = 6) did not use typical areas of anterior activation [dorsolateral prefrontal cortex (DLPFC)] and showed less anterior activity than did controls (*n* = 6) without solvent exposure but with comparable behavioral performance on the experimental task. That study, however, included a small number of subjects (*n* = 6) diagnosed with cognitive dysfunction based on a previous comprehensive neuropsychological evaluation and therefore may not reflect the larger group of workers chronically exposed to solvents.

Thus, the purpose of the present study was to evaluate neural activation patterns with functional MRI (fMRI) during performance of working memory tasks among construction workers with and without chronic exposure to solvents and without a diagnosed cognitive impairment.

## Materials and Methods

*Subjects.* One hundred and thirty-three solvent-exposed subjects (industrial painters) and 78 controls (drywall tapers, glazers, carpenters) were recruited for the study, which included a screening medical evaluation; a structured psychiatric interview (Composite International Diagnostic Interview for *Diagnostic and Statistical Manual of Mental Disorders*, Fourth Edition [DSM-IV (CIDI)] (American Psychiatric Association 1994); cognitive and sensory testing; assessment of lifetime use of alcohol, marijuana, and cocaine; and lifetime history of solvent exposure. A subgroup of these subjects volunteered to participate in the fMRI study (46 solvent exposed; 39 controls). There were no females among the participants. Solvent-exposed workers had at least 10 years of employment in their respective trades. Control subjects who reported solvent exposure were excluded (*n* = 4). After screening for psychiatric conditions, current medication use, MRI compatibility, incidental findings, ability to perform the N-Back task, and excessive motion during the scans, 19 solvent-exposed workers and 12 controls were eliminated from further analysis. For the depression assessment, we used the Beck Depression Inventory-II (BDI-II) questionnaire, a widely used depression scale with excellent reliability (Beck et al. 1996). Reading test scores [North American Adult Reading Test (NAART) (Blair and Spreen 1989)] were used as an indicator of verbal intelligence. After the study procedures were fully explained, all participants provided informed consent. Study protocols, including informed consent procedures, were approved by the institutional review boards of UMDNJ-Robert Wood Johnson Medical School and Mount Sinai Medical School.

*Exposure assessment.* We calculated a cumulative lifetime exposure index for each subject who reported ever working with solvent-based paints. The work duration and time spent performing specific job tasks (spray, roller, brush, rag/sponge, and cleaning equipment) was determined based on the completion of a computerized questionnaire administered by a trained technician. Subjects reported painting activities in 5-year intervals, estimating the percentage of time spent in each activity and the protective equipment worn during those activities for their working lifetime. Subjects reported in the questionnaire that they uniformly wore clothing that covered their skin during bridge and other industrial painting tasks, so no estimate of dermal exposure from solvents was included in the final exposure calculation.

To assess representative air concentrations of solvents during current painting activities, field samples were collected in a series of week-long sampling programs during different seasons at New Jersey Department of Transportation and New York City Bridge Maintenance work sites (Qian et al. 2010). Historic estimates of exposure to solvents were estimated from changes in paint composition determined by U.S. Environmental Protection Agency (EPA) regulations to reduce ozone, research literature reports from the past 25 years (e.g., PubMed, TOXLINE), regulatory documents (e.g., Health Hazard Evaluation, U.S. EPA, Material Safety Data Sheets), and commercial sources (e.g., National Paints and Coatings Association, *Journal of Protective Coatings and Linings*). We followed this approach because only limited data exist documenting historical air concentrations of organic solvents during construction painting.

Exposure to solvents during painting activities, however, is reduced with the use of protective equipment. For example, since 1998 painters have been required by the Occupational Safety and Health Administration (OSHA) to wear respirators when spraying in a confined space (OSHA 1998) but not for brushing or rolling operations. Therefore, lifetime exposure to solvents was modified based on the type of respirator used and the amount of time a respirator was worn during different painting activities. We searched the American National Standards Institute, National Institute for Occupational Safety and Health (NIOSH), OSHA, PubMed (National Center for Biotechnology Information 2010), TOXLINE (National Library of Medicine 2010), and respirator manufacturers’ databases for current and historical assigned protection factors of different respirators.

The solvent exposure index was calculated from the time spent applying paint with each application method (*T*), air concentrations of organic solvents for that application method during the appropriate time period (*C*), and a protection factor for the protective equipment (*P*) used during the appropriate time period according to Equation 1:


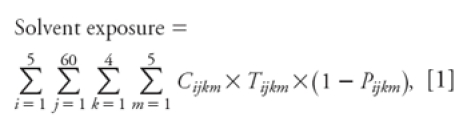


where *i, j, k*, and *m* are indices for the five 5-year intervals worked (1981–2005), months worked in each 5-year interval, weeks per month, and application method, respectively (questionnaire data); *C_ijkm_* is the air concentration of organic solvent (parts per million; personal air sampling badges, field air samples, historical paint composition, and air concentration data); *T_ijkm_* is the painting time (hours per week; questionnaire data); and *P_ijkm_* is the protection factor of respirator type (questionnaire data and historical protection factors).

The calculated solvent exposures were summed over all the exposure events of the subject’s career to generate the cumulative lifetime solvent exposure index in parts per million-hours. We performed a Monte Carlo simulation using 240 iterations to provide an empirical distribution of the exposure simulation for each painter, rather than calculating the exposure index as a single mean concentration for each painting method and protection factor for all painters during each time period. The median value from the distribution was assigned as the exposure index for each subject. For the Monte Carlo simulation, the time spent painting and the protective equipment used were entered as constants for each 5-year time period based on questionnaire responses (Wang et al. in press). Air concentrations varied using the actual data collected during current painting activities (Qian et al. 2010), adjusted with data from the literature documenting historic air concentrations.

*Imaging.* All imaging was performed on a 1.5-T Sonata MRI scanner (Siemens, Erlangen, Germany). We performed fMRI using a blood-oxygenation level–dependent (BOLD) technique, an MRI method to measure regional blood flow (Ogawa et al. 1990). Gradient echo planar imaging sequence was acquired using the following protocol: 32 axial slices, 3 mm skip 1 mm, repetition time (TR) = 2.5 sec, echo time (TE) = 30 msec, flip angle = 90°, field of view (FOV) = 21 cm, matrix size = 64 × 64. For incidental pathology screening, we also acquired T2-weighted anatomical scans of the whole brain using a turbo spin-echo pulse sequence (34 axial slices, TR = 5,380 msec, TE = 99 msec, flip angle = 170°, FOV = 210 mm, matrix = 512 × 336, voxel size = 0.41 × 0.41 × 4 mm). All scans were read by a radiologist for incidental findings. For coregistration and normalization purposes, a high-resolution T1-weighted structural image with good gray/white matter contrast was acquired using a three-dimensional  magnetization-prepared rapid-gradient echo sequence with the following protocol: matrix size = 256 × 256 × 208, FOV = 21 cm, TR = 2,500 msec, TE = 4.38 msec, inversion time (TI) = 1,100 msec, and an 8° flip-angle fast low-angle-shot (FLASH) acquisition, giving a total imaging time of about 10 min.

*Working memory task.* We applied an N-Back task during the fMRI acquisition for activation of the working memory circuitry. The N-Back task (Kirchner 1958; Owen et al. 2005) is a continuous performance task that is commonly used in functional imaging for the study of cognition where memory load can be adjusted using the parameter N. In our implementation of the fMRI-compatible N-Back task, the subject is presented with a sequence of letters (using a visual display mounted in the MRI scanner bore) and asked to press a button when presented with target letters defined according to one of N prespecified conditions (in this case, four conditions referred to as 0-, 1-, 2-, or 3-back). For the 0-back condition, the subject had to identify the target letter “T” each time it was presented. For the 1-back condition, the subject was asked to identify each letter that matched the letter preceding it, so that target letters for the sequence “B*DD*O*FF*HB*YY*F*KK*L,” for example, would have been the second D, F, Y, and K in the sequence (shown in italics for illustration only). For a 2-back pattern the subject was asked to identify letters that matched a letter two positions back in the sequence (e.g., the second K, F, U, and S in the sequence “D*K*J*KF*G*FU*B*U*C*S*K*S*”), and for a 3-back pattern the subject was asked to identify letters that matched a letter three positions back. Subjects received instructions and performed testing trials for each condition before they entered the MRI suite. Each of the four conditions was repeated three times in a counterbalanced pseudo-random order, with each 28-sec N-Back run preceded by an 8-sec period of fixation and a 4-sec instruction period to inform the subject of the task level (0-, 1-, 2-, or 3-back; total scan time, 8 min 8 sec). Responses were recorded using a Brainlogics fiber optics subject response glove (PST Inc., Pittsburgh, PA). Task performance was scored using the ePrime program (PST Inc.) as correct hits (subject identified the correct target), false hits (subject pressed the button when a nontarget letter was displayed), and misses (subject did not press the button when a target letter was displayed). In addition, the 0-back condition was used to determine average reaction times by recording the time difference between the presentation of the 0-back target and the time of button press.

*Analysis.* For analysis and visualization of the fMRI, we used both software developed in-house under Matlab (version 7.8; Mathworks Inc., Natick, MA) and public domain software. BOLD data were processed using SPM5 software (Wellcome Department of Cognitive Neurology, Institute of Neurology, University College London, London, UK). fMRI analysis followed standard procedures using SPM5. Briefly, functional data was slice-time corrected by interpolation to the middle slice before motion correction. The functional images were coregistered to each subject’s anatomical scans. Coregistered anatomical images were then segmented to produce the parameters used for normalization into a standard anatomical brain reference template developed by the Montreal Neurological Institute (Collins et al. 1994; Evans et al. 1992). Using this reference atlas, voxel locations in the brain can be referenced using absolute x-, y-, and z-coordinates. The number of voxels in an activated region is indicated as cluster size. The brain region that such voxel/coordinates fall into is indicated by a Brodmann area number, as is normally done in fMRI reports (Brodmann 1909). We have also indicated the actual names of these regions [e.g., anterior cingulate cortex (ACC), DLPFC, insular cortex IC, and parietal portex (PC)]. Images were spatially smoothed using a 6-mm isotropic Gaussian smoothing kernel. Individual contrast images were produced in the context of the general linear model using a boxcar function (0-back vs. 1-, 2-, 3-back) convolved with a canonical hemodynamic response function. Next, to produce a group activation map, we performed a one-sample *t*-test using each subject’s contrast images to determine areas of increased activity during the active (1-, 2-, 3-back) state compared with baseline (0-back). Significant clusters were identified (*p* < 0.001, uncorrected). Additional voxelwise *t*-tests for group differences in activation were computed (Toga and Mazziota 2002). A voxelwise analysis of covariance (ANCOVA) was computed to compare the activation levels of each group while accounting for differences in overall task performance using d-prime as a nuisance variable. For the solvent-exposed group, we also computed voxel-by-voxel correlation maps between percent activation (defined as the amount of BOLD signal level while performing the N-Back task relative to the BOLD signal while at rest) and solvent exposure levels. To analyze regional blood flow differences, we extracted percent activation values from the regions as defined in the significant activation clusters in the group maps. Regions of interest (ROIs) with 3-mm radii were obtained from the local maxima of these clusters. Percent activation values for these ROIs were extracted from individual subjects’ scans and transferred to Statistica (version 9; Statsoft Inc., Tulsa, OK) for further statistical analysis. Partial correlation analysis was performed while controlling for the following confounding variables: current blood lead level; verbal IQ (NAART); lifetime alcohol, marijuana, and cocaine use; age; and depression score. NAART and lifetime alcohol use, lead exposure, marijuana use, and cocaine use for each subject were classified by percentile rank according to the distribution among all study participants.

*Behavioral.* We used *t*-tests to compare N-Back accuracy and reaction time between solvent-exposed subjects and controls.

## Results

Among the painters, we excluded 19 subjects from the analysis because of current psychiatric disorder (*n* = 3), current use of medication (*n* = 3), no solvent exposure (*n* = 5), and incomplete data (*n* = 8). Among the controls, we excluded 12 because of exposure to solvents (*n* = 4), current psychiatric disorder (*n* = 1), medication use (*n* = 2), anatomical finding (*n* = 1), and incomplete data (*n* = 4). Solvent-exposed subjects were similar to controls in age; education; number of years worked; lifetime alcohol, marijuana, and cocaine use; and BDI scores (Table 1). Although from similar occupational groups, the solvent-exposed subjects had significantly lower reading test scores (NAART) and significantly higher blood lead concentrations than did controls. Controls were more likely to be Caucasian than were solvent-exposed workers.

**Table 1:** Demographics of subjects who completed the fMRI study.

Table 1. Demographics of subjects who completed the fMRI study.						
Variable		Solvent exposed (*n* = 23)		Controls (*n* = 27)		*p*-Value
Age (years)		46.22 ± 1.62		45.85 ± 1.48		0.8683
Education (years)		12.52 ± 0.36		12.5 ± 0.24		0.9593
Race/ethnicity						
Caucasian		17 (74)		25 (93)		
Hispanic		6 (26)		2 (7)		
Other		0		0		
Verbal IQ (NAART)		93.37 ± 2.07		101.93 ± 1.30		0.0007*
Years worked		21.05 ± 1.46		21.48 ± 1.22		0.8192
Blood lead (μg/dL)		5.91 ± 0.82		2.48 ± 0.20		0.0001*
Solvent exposure (10^6^ ppm-hr)		3.89 ± 1.02		0		0.0001*
Lifetime alcohol use (percentile rank)		34.43 ± 5.44		44.74 ± 4.05		0.1289
Lifetime marijuana use (percentile rank)		34.83 ± 6.13		33.48 ± 5.68		0.8729
Lifetime cocaine use (percentile rank)		24.09 ± 6.21		33 ± 6.64		0.3373
BDI depression total		2.43 ± 0.54		3.19 ± 0.72		0.4211
BDI cognitive		0.35 ± 0.13		0.67 ± 0.21		0.2312
BDI somatic/affective		1.3 ± 0.3		1.44 ± 0.33		0.7557
Values shown are mean ± SE or *n* (%). *Statistically significant by *t*‑test.						

The performance scores of the N-Back task (Table 2) showed that solvent-exposed subjects scored significantly fewer correct hits (*p* = 0.005) and more false hits (*p* = 0.016) than controls, consistent with previous neurobehavioral studies (Bockelmann et al. 2002; Colvin et al. 1993; Kishi et al. 1993; Morrow et al. 1992; Ryan et al. 1988). For the 0-back condition (press button when target appears) used to estimate reaction time, solvent-exposed subjects were slower than controls (*p* = 0.034).

**Table 2:** N‑Back behavioral performance results (mean ± SE).

Table 2. N‑Back behavioral performance results (mean ± SE).						
N‑Back performance variable		Exposed (*n* = 23)		Control (*n* = 27)		Significance
Correct hits*a*		21.43 ± 1.26		26.41 ± 1.14		*t*_48_ = 2.9257
						*p* = 0.0052
False hits		14.74 ± 3.17		6.82 ± 1.14		*t*_48_ = –2.4986
						*p* = 0.0159
Response time (msec)		333.47 ± 41.88		232.68 ± 23.33		*t*_48_ = –2.1843
						*p* = 0.0339
**a**Total number of targets, 36.						

**Figure 1:**
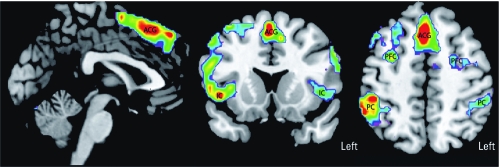
SPM group activation map comparing N‑Back performance with rest for all subjects [painters (*n* = 27) and controls (*n* = 27)]. Significant activated clusters (*p* < 0.001) in the anterior cingulate gyrus (ACG), prefrontal cortex (PFC), IC, and PC are shown using a rainbow color spectrum superimposed on anatomical magnetic resonance images. Significantly activated voxels are colored from purple (*p* = 0.001) to red (variable depending on location, but at least *p* < 0.001).

**Figure 2:**
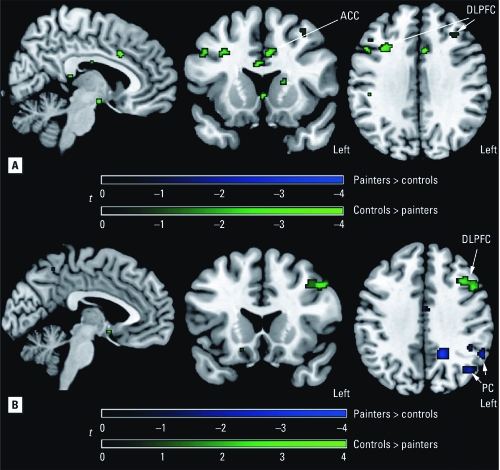
SPM maps corrected for verbal IQ and lead exposure (*A*) or corrected for verbal IQ, lead exposure, and task performance (*B*) of significant group differences (*p* < 0.001) in activation of the working memory network. Green indicates regions where painters had lower activation and blue indicates regions where painters had higher activation, compared with controls.

**Figure 3:**
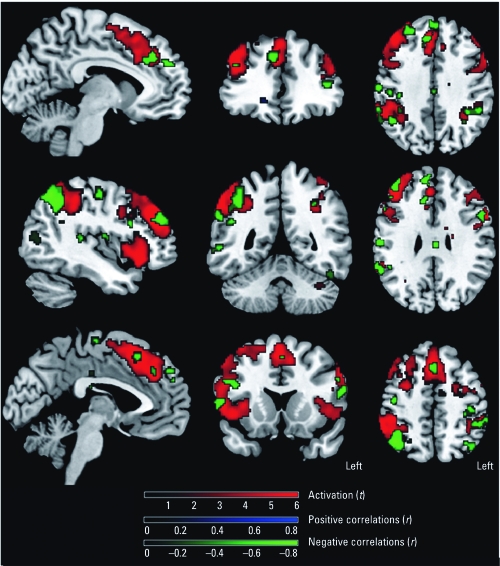
SPM maps of significant correlations (*p* < 0.001) between activation and lifetime solvent exposure in painters. Green indicates decreased activation with increased exposure levels (observed in anterior cingulate gyrus, prefrontal cortex, PC, and IC); blue indicates increased activation with increased exposure levels (no significant voxels present); and red indicates general group activation patterns with the N‑Back task.

The statistical parametric mapping (SPM) group maps show areas of activation consistent with other fMRI studies using working memory paradigms in normal controls. We detected significant activated clusters in the ACC, DLPFC, PC, and insular cortex (IC) (Figure 1, Table 3). Because of the imbalance in race (no African Americans in the control group), we performed the between-group comparisons on Caucasians only. After correcting for verbal IQ and lead exposure, SPM maps showed that, relative to controls, solvent-exposed subjects had reduced activation in areas in the ACC and bilateral DLPFC (Figure 2A). Accounting for task performance differences, the ANCOVA showed that the solvent-exposed subjects had significantly reduced activation in the left DLPFC and increased activation in the left parietal regions compared with controls (Figure 2B). Among solvent-exposed subjects, lifetime solvent exposure was significantly and negatively correlated with activation detected in the ACC, DLPFC, and PC after control for confounders (Figure 3, Table 4). Percent activation values extracted from the ROIs also showed significant correlations with lifetime solvent exposure (Table 4).

**Table 3:** Brain regions with activation that showed a significant correlation with the N‑Back task for the group (controls and painters combined).

Table 3. Brain regions with activation that showed a significant correlation with the N‑Back task for the group (controls and painters combined).								
Brain region		MNI coordinates		Brodmann area		Cluster size		*t*-Value*
ACC		4, 22, 44		24		1,322		7.8558
L-DLPFC		–58, 12, 30		46		203		6.4715
R-DLPFC		44, 36, 34		46		980		7.7174
L-PC		–48, –40, 50		40		32		4.6374
R-PC		58, –46, 40		40		1,198		8.0981
L-IC		–34, 20, 8		44		302		6.9214
R-IC		42, 24, –8		44		1,214		8.8248
Abbreviations: L, left; MNI, Montreal Neurological Institute; R, right. *All *p* < 0.0001.								

**Table 4:** Partial correlations in solvent-exposed subjects between percent activation and level of solvent exposure (controlling for lead exposure; verbal IQ; lifetime alcohol, marijuana, and cocaine use; age; and BDI scales).

Table 4. Partial correlations in solvent-exposed subjects between percent activation and level of solvent exposure (controlling for lead exposure; verbal IQ; lifetime alcohol, marijuana, and cocaine use; age; and BDI scales).														
				DLPFC				PC				IC		
Measure		ACC		Left		Right		Left		Right		Left		Right
*r*-Value		–0.68		–0.55		–0.57		–0.64		–0.30		–0.26		–0.12
*p*-Value		0.001		0.013		< 0.009		0.002		0.201		0.262		0.622

## Discussion

This is one of the few human fMRI studies to evaluate activation patterns among individuals with chronic occupational exposure to solvent mixtures and control of potential confounders. As hypothesized, our study showed a negative correlation between solvent exposure and activation in the ACC, DLPFC, and PC among the solvent-exposed group, suggesting defects in the brain circuitry underlying performance of attention and working memory tasks. Moreover, solvent-exposed subjects exhibited significantly lower activation of the ACC and DLPFC and significantly worse performance on the N-Back task than did controls. Our findings of lower activity in the ACC and DLPFC of solvent-exposed workers compared with controls is consistent with studies that used nuclear tracer techniques such as single-photon-emission computed tomography (SPECT) or PET and found reduced blood flow in these areas (Callender et al. 1993; Fincher et al. 1997; Haut et al. 2000).

The behavioral performances between solvent-exposed workers and controls were significantly different, and one might argue that the resulting activation differences are the result of performance. We ran a voxel-by-voxel ANCOVA to take into account the behavioral performance differences between the groups. This result showed that the left DLPFC had reduced activation in the solvent-exposed subjects but regions in their left PC showed increased activity relative to the controls. Evidence of compensatory increases in the function of regions in the PC was also reported in a PET study by Haut et. al. (2000). Although in that study a much smaller number of subjects were imaged (six exposed to organic solvents vs. six controls) using a different working memory task, and the authors also reported reduced activation in the left DLPFC. One explanation for this left-sided significance is that our working memory task involved letters and thus was likely to be verbally mediated (Petrides et al. 1993; Smith et al. 1996).

It has been widely accepted that working memory is supported by a tightly integrated network of the frontal and parietal regions (Chafee and Goldman-Rakic 2000; Jung and Haier 2007; Posner and Petersen 1990). A dysfunctional prefrontal cortex may cause aberrant activation patterns in the posterior aspects of the brain. Our finding of increased activity in the parietal lobe in solvent-exposed subjects compared with controls supports this notion. Some fMRI studies have shown a population sample having hyperactivity in the target brain region as an indicator of dysfunction. This hyperactivity is assumed to be an indicator of local compensatory function: The less “efficient” brain region has to work harder (showing increased blood flow) to perform the same task, as has been shown in studies of normal cognition and intelligence (Haier 1993; Neubauer and Fink 2009). Other fMRI studies have detected a different pattern of activation in the population sample, which is taken as an indicator of brain plasticity where other brain regions have taken over the function of the target brain region, which is allegedly defective. This has been reported in alcohol studies (Pfefferbaum et al. 2001) and in solvent exposure studies (Haut et al. 2000). The observed hypoactivity is similar to studies that found reduced activation in areas affected by traumatic brain injury (Chen et al. 2004; McAllister et al. 2006) or Alzheimer’s disease (Johnson et al. 2006). In the present study we found this reduced activity in the DLPFC to be more specific in the left DLPFC after we accounted for the task performance. The reduced activation in the affected brain areas is consistent with altered neuronal pathology, which might be caused by an altered blood supply mechanism or neuronal death due to solvent exposure rather than due to performance.

As part of the present study, we also acquired proton density and T2-weighted images for the purpose of screening for incidental pathologies. We excluded from the analysis patients with white matter abnormalities, as determined by hyperintensities in the T2-weighted images. This suggests that the loci that differed between the solvent-exposed subjects and the controls involved gray matter regions, although we cannot rule out the possibility that white matter played a role as well. A study using other imaging techniques such as diffusion tensor imaging (Basser et al. 2000) might elucidate the role of white matter. Our correlation results also show an inverse relationship between activation and lifetime solvent exposure among the solvent-exposed subjects, supporting a direct relationship between exposure and blood flow in brain regions that subserve working memory as opposed to indirect compensatory or other cognitive effects.

The prefrontal cortex together with the parietal and anterior cingulate forms the neural substrate for working memory and attention (Posner and Petersen 1990; Smith and Jonides 1999; Smith et al. 1998). The prefrontal cortex is the last brain region to develop in the human brain and may be the most vulnerable to physical or chemical insults. The prefrontal cortex is the most vulnerable in neurodegenerative disorders, and atrophy of the prefrontal cortex is also one of the morphological changes in aging (Decarli et al. 1994; Fuster 2008; Raz et al. 1997).

A few studies have looked into solvent effects on neurotransmitters such as dopamine, acetylcholine, and γ-aminobutyric acid (GABA), with limited consistent results. Brain dopamine concentrations with solvent exposure have been studied in rodent models, and both increases (Rea et al. 1984) and decreases (Kondo et al. 1995) have been reported, albeit using different methodologies. The dopamine relationship is certainly relevant considering that it is an important neurotransmitter involved in DLPFC signaling (Williams and Goldman-Rakic 1995). Increases in striatal acetylcholine with exposure levels have also been reported (Honma 1983; Stengard 1995; Tsuga and Honma 2000). Changes in GABA binding detected in rat frontal cortex depended on the chronic or subchronic exposure levels (Beckstead et al. 2000; Bjornaes and Naalsund 1988). GABA might be a reasonable target considering the addictive effects in the recreational use of certain solvents. Many additional studies on the effects of solvents on other neurotransmitter actions such as GABA_a_, glycine, *N*-methyl-d-aspartic acid, nicotine, and 5-hydroxytryptamine-3 have been performed (Bowen et al. 2006).

Anatomical studies of solvent exposure in humans have shown diffuse atrophy in the cerebellar regions, brainstem, frontal cortex, and PC (Keski-Santti et al. 2009; Rosenberg et al. 2002; Thuomas et al. 1996). Most of these anatomical findings were nonspecific, and some included the frontal and parietal regions where we found evidence of functional deficits. The results of our functional imaging study in the frontal and parietal regions are also consistent with the diminished cognitive function observed among solvent-exposed subjects.

The solvent-exposed subjects in our study have worked with solvent mixtures for an average of 22 years. Unfortunately, we do not have actual historical measurements of solvent exposure for our subjects and therefore cannot determine a specific dose–response analysis for the imaging results in this study. Nevertheless, the highly significant relationship between our lifetime solvent exposure metric and activation patterns validates the neural basis for alterations in cognitive function so often observed among these workers. Future studies are needed to better understand the exposure concentrations over a working lifetime associated with neurological effects among workers.

The current OSHA permissible exposure limit (PEL) for toluene is 200 ppm, the same value as for general industry. The (NIOSH) PEL is 100 ppm, whereas the threshold limit value for toluene as recommended by American Conference of Governmental Industrial Hygienists (ACGIH) is 50 ppm (Agency for Toxic Substances and Disease Registry 2001). Unfortunately, without actual historical exposure measurements for our subjects, we cannot be certain of the exact solvent concentrations to which they were exposed. Although many manufacturing sites follow current and historical ACGIH recommendations, worksites for construction painters are often less well controlled, and industrial hygiene measurements are not routinely collected (Qian et al. 2010). Therefore, excursions from recommended solvent exposure limits may have occurred. In addition, it is not unreasonable to believe that our sample of solvent-exposed subjects was exposed to higher concentrations historically than those recommended currently by ACGIH based on their average (i.e., 22 years) and minimum (10 years) lengths of time spent working with solvent-based paints. Under these estimated exposure conditions, our results show changes in the biological substrates that are consistent with the neurobehavioral observations documented in an extensive literature of workers who continue to be exposed to solvent mixtures during their working lifetime.
